# Mining Potential Drug Targets and Constructing Diagnostic Models for Heart Failure Based on miRNA-mRNA Networks

**DOI:** 10.1155/2022/9652169

**Published:** 2022-09-27

**Authors:** Xiangming Fang, Rensheng Song, Jiaxing Wei, Qin Liao, Zhenhong Zeng

**Affiliations:** ^1^Key Laboratory of Biological Targeting Diagnosis, Therapy and Rehabilitation of Guangdong Higher Education Institutes, The Fifth Affiliated Hospital of Guangzhou Medical University Intensive Care Unit, Guangzhou, China 510700; ^2^Key Laboratory of Biological Targeting Diagnosis, Therapy and Rehabilitation of Guangdong Higher Education Institutes, The Fifth Affiliated Hospital of Guangzhou Medical University Cardiovascular Medicine Department, Guangzhou, China 510700

## Abstract

Heart failure (HF) is a globally prevalent cardiovascular disease, but effective drug targets and diagnostic models are still lacking. This study was designed to investigate effective drug targets and diagnostic models for HF in terms of miRNA targets, hoping to contribute to the understanding and treatment of HF. Using HF miRNA and gene expression profile data from the GEO database, we analyzed differentially expressed miRNAs/gene identification in HF using Limma and predicted miRNA targets by the online TargetScan database. Subsequently, gene set enrichment analysis and annotation were performed using WebGestaltR package. Protein-protein interactions were identified using the STRING database. The proximity of drugs to treat HF was also calculated and predicted for potential target therapeutic drug. In addition, further drug identification was performed by molecular docking. Finally, diagnostic models were constructed based on differential miRNAs. The GEO dataset was used to screen 66 differentially expressed miRNAs, incorporating 56 downregulated miRNAs and 10 upregulated miRNAs. The JAK-STAT signaling pathway, MAPK signaling pathway, p53 signaling pathway, Prolactin signaling pathway, and TGF-beta signaling pathway were enriched, as shown by KEGG enrichment analysis on the target genes. In addition, we found that 83 genes were upregulated and 92 genes were downregulated in HF patients vs. healthy individuals. Based on the inflammation-related score, hypoxia-related score, and energy metabolism-related score, we identified key miRNA-mRNA pairs and constructed an interaction network. Following that, TAP1, which had the highest expression and network connectivity in acute HF with crystal and molecular docking studies, was selected as a key candidate gene in the network. And the compound DB04847 was selected to produce a large number of favorable interactions with TAP1 protein. Finally, we constructed two diagnostic models based on the differential miRNAs hsa-miR-6785-5p and hsa-miR-4443. In conclusion, we identified TAP1, a key candidate gene in the diagnosis and treatment of HF, and determined that compound DB04847 is highly likely to be a potential inhibitor of TAP1. The TAP1 gene was also found to be regulated by hsa-miR-6785-5p and hsa-miR-4443, and a diagnostic model was constructed. This provides a new promising direction to improve the diagnosis, prognosis, and treatment outcome and guide more effective immunotherapy strategies of HF.

## 1. Introduction

Heart failure (HF) results from the dysfunction of diastolic and/or the systolic function of the heart; insufficient blood perfusion and blood stasis in the venous system in the arterial system would occur if the venous return blood could not be fully discharged from the heart, thereby causing cardiac circulatory disorder syndrome [1, 2]. Instead of an independent disease, HF is a terminal stage in heart disease development. A great number of HF begins with left HF, which first manifests as pulmonary circulation congestion. Advanced interventions such as drug therapy, cardiac synchronization therapy, and heart transplantation mainly focus on the control of heart failure-related symptoms [3] Mortality in HF could be reduced to some extent through these interventions [4]. Nevertheless, the discovery of new drugs and new diagnostic strategies is still necessary to further reduce mortality and improve quality of life in the process of HF. Therefore, further research and in-depth understanding of the biological mechanisms of HF are urgently needed.

In a healthy heart, 90% of ATP production is produced via mitochondrial oxidative phosphorylation, and 60–70% of the energy was derived from lipid oxidation [5]. Therefore, the heart is greatly dependent on the continuous supply of fatty acids and oxygen. Moreover, under diverse and nonoptimal physiological conditions, the heart has great metabolic plasticity and could maintain ATP production, using other substrates such as amino acids, glucose, ketone bodies, and lactate [6]. However, a combination of oxidative and substrate level phosphorylation more evenly produces skeletal muscle ATP, allowing a relatively more flexibility in terms of oxygen demand and substrate use [7]. A heart deprived of oxygen shows a decreased ATP production and mitochondrial respiration [8]. Under hypoxia, a significant loss of mitochondrial density and skeletal muscle mass [9] could be seen as an adaptive modification that lowers reactive oxygen species (ROS) and reduces tissue's demand for low O_2_ [9].

At the posttranscriptional level, microRNAs (miRNAs) with approximately 22 nucleotides regulate expression of genes [10, 11]. Such a process involves binding to the complementary sequence of messenger RNA (mRNA), subsequently resulting in degradation of the mRNA or translational inhibition [12]. Study showed that miRNAs play a role in multiple pathophysiological mechanisms, including in HF development [13]. In the circulation, extracellular miRNAs are measurable, and they have now been increasingly regarded as prognostic and diagnostic biomarkers in various diseases [14]. As a new category of biomarkers, studies have shown the potential of miRNAs in HF [15], which are critical drivers of cardiac tissue remodeling and can be used as therapeutic targets. For example, in cardiac hypertrophic remodeling, miR-132 has been previously verified as a master switch [16] and is markedly increased in the early hypertrophic phase of HF [17], and in genetic or pharmacological studies, inhibition of miR-132 has the effect of reversing or preventing the progression of HF [18]. Transcription factor 3 (ATF3) expression was increased in human hypertrophic heart; ATF3 upregulation protects the heart by suppressing Map2K3 expression and subsequent p38-transforming growth factor-*β* signaling [19]. Therefore, analysis and exploration of miRNAs-mRNA as potential drug targets for the treatment of HF have great potential.

The heart, as a vital tissue that maintains blood circulation, ensures the metabolic needs. And it is too hard to obtain the tissue, especially in a healthy body. Based on the above statements, this study is aimed at mining key drug targets in HF using the NCBI Gene Expression Omnibus (GEO) dataset, including expression profiles of mRNAs and miRNAs. Differential miRNA and differential gene expression profiles of healthy controls and HF subjects were analyzed with the “Limma” package in R software. After that, we detected the key target gene of key miRNA, namely, TAP1. Finally, a diagnostic model was constructed based on the differential miRNAs. The current findings contributed to the development of understanding novel molecular mechanisms of HF pathogenesis, particularly the possible association of dysregulated pathways of the TAP1 gene with HF pathological processes, and ultimately predicted a new therapeutic target drug DB04847 for HF patients.

## 2. Methods

### 2.1. Data Collection and Preprocessing

Expression profile data were downloaded from the NCBI GEO database [20] for miRNA number GSE104150, which contained a total of 2549 miRNAs from 7 healthy controls and 9 patients with HF. mRNA expression profile data were downloaded from the NCBI GEO database for mRNA number GSE21125, which contained 9 patients with acute HF, 9 patients with chronic HF, 9 patients at risk of HF, 9 patients with left ventricular dysfunction, and 9 healthy controls. In this analysis, we kept only 9 patients with acute HF, 9 patients with chronic HF, and 9 healthy controls, for a total of 18 HF patients, 9 healthy controls, and 20,295 genes.

HF with other diseases was excluded, and HF with survival information was retained.

### 2.2. Analysis of Differentially Expressed miRNAs/Genes and Functional Enrichment

Differentially expressed miRNAs were analyzed using Limma [21] and filtered using the criteria of |log2(fold change)| > 1 and FDR (false discovery rate) < 0.05. Similarly, differentially expressed genes were analyzed using Limma and filtered using the criteria of |log2(fold change)| > 1 and *P* < 0.05. We performed Kyoto Encyclopedia of Genes and Genomes (KEGG) enrichment analysis on miRNA target genes by the R software package clusterProfiler, filtered at *P* value < 0.05.

We downloaded energy metabolism-related pathways from the Gene Set Enrichment Analysis (GSEA) [22] website for subsequent enrichment analysis and downloaded genes related to Toll-like receptor signaling pathway, NF-kappa B signaling pathway, JAK-STAT signaling pathway, T-cell receptor signaling pathway, INFLAMMATORY_RESPONSE, B-cell receptor signaling pathway, and other associated pathways. KEGG enrichment analysis of differential genes was performed by WebGestaltR package. The enrichment scores of each pathway in the KEGG pathway regarding the samples were calculated by the GSVA package, and the correlation between genes and pathways was calculated by the rcorr function of the Hmisc package; here, we used *P* < 0.05 and cor > 0.4 as the threshold. In addition, we also performed single-sample gene set enrichment analysis (ssGSEA) of KEGG-related pathways by the R language GSVA package and analyzed pathways that were statistically significant in HF patients and healthy individuals by *t*-test (*P* < 0.05).

### 2.3. miRNA Target Analysis

The miRNA regulatory relationships were predicted from the online TargetScan [23] (https://www.targetscan.org/vert_72/) regulatory database. TargetScan is a software for predicting miRNA binding sites in mammals. Before prediction of miRNA target genes, the 3′UTR region of the transcript needs to be determined first. The TargetScan database identifies the corresponding 3′UTR region of the transcript by a sequencing technique called 3P-seq (miRNAs in mammals bind the 3′UTR region of the transcript sequence to exert posttranscriptional regulation) and provides a comprehensive sequence of the 3′UTR region by combining the analysis results of this technique with the available 3′UTR annotations in NCBI.

### 2.4. Protein-Protein Interaction Network Construction

The STRING (https://string-db.org/) database [24] supports the search for known protein-protein interactions and predicted protein-protein interactions (PPI). The PPI database can be applied to 2031 species and contains 9.6 million proteins and 13.8 million types of protein. In addition to results predicted using bioinformatics methods, it stores results of text mining from PubMed abstracts and synthesis of data from other databases as well as experimental data. Exploring interaction networks among proteins helps to mine the core regulatory genes. Though already many protein interaction databases are available to us, STRING is one of them covering the most species with the largest interaction information. We used STRING web version (version v11.0, https://string-db.org/) in this study, and based on the STRING database of protein interaction relationships, we filtered the interaction score≧400, that is, medium confidence.

### 2.5. Prediction of Potential Target Therapeutic Agents

We calculated the proximity of drugs and its effect on treating HF. Here, we can give *S* (the gene set related to the treatment of heart failure), *T* (the set of drug target genes), *D* (the degree of the node of the related gene set in the PPI), and the distance *d*(*s*, *t*), which indicated the shortest path between node *t* and node *s* (where *s* ∈ *S*, HF-related genes; *t* ∈ *T*, drug target genes). The calculation was as shown below:

(1) $$\begin{array}{c} d\left(S,T\right)=\frac{1}{\left|\;T\right|}\underset{t\in T}{\sum }\min_{s\in S}\left(d\left(s,t\right)+\omega \right), \end{array}$$

where *ω* is the weight of the target gene. The calculation method is *ω* = −ln (*D* + 1), otherwise if the target gene is a gene in the HF-related gene set, *ω* = 0.

We calculated simulated reference distance distribution corresponding to the drug. Protein nodes were randomly seen as the simulated drug target in the network, with the number of nodes keeping the same as the target scale (denoted as *R*). The distance *d*(*S*, *R*) between these simulated drug targets (representing simulated drugs) and the TAP1-related gene set was then calculated, and after 10,000 random repetitions, a simulated reference distribution was calculated. Using both *μd* (*S*, *R*) and *σd*(*S*, *R*), the mean and standard deviation of the corresponding actual observation distance and the reference distribution were converted into a standardized score, that is, the degree of proximity *z*:

(2) $$\begin{array}{c} z\left(S,T\right)=\frac{d\left(S,T\right)-\mu_{d\left(S,R\right)}}{\sigma_{d\left(S,R\right)}}. \end{array}$$

We found that whether we took the TAP1-related gene set as the sample or our randomly selected gene set as the sample, in the location of central distribution of the drug distance, we performed multiple hypothesis tests with the random data obtained in the reference and selected drug with short distance and FDR < 0.001; a candidate drug set related to the TAP1-related gene set was obtained as an analysis.

### 2.6. Molecular Docking

AutoDock Vina software [25] was used for molecular docking. Firstly, to prepare all the input files, AutoDockTools 1.5.6 was used. The PDB files of the proteins were downloaded from the Protein Data Bank (PDB ID: 6SUK). All protein B chains, water molecules, and potassium ions were removed, and polar hydrogens were added to the system. The charge of the zinc ion in the PDBQT file of the receptor protein was changed to +2.0. The coordinates of the grid in each *XYZ* direction during molecular docking were 20.2 Å, -46.5 Å, and 15.2 Å. The lengths in each *XYZ* direction were 20 Å. To identify the most binding mode of the ligand molecule, the Lamarckian algorithm was introduced. The maximum number of conformations of output was set to 10, the exhaustiveness was set to 8, and the maximum energy range allowed was set to 3 kcal/mol. The processing of the result maps was performed using PyMOL.

The following are used for conditional screening: (1) Homo sapiens, (2) resolution: 1.75 Å, (3) ligand: dual inhibitor of omapatrilat metalloproteinases ACE and NEP with Ki values of 0.64 nM and 0.45 nM, respectively, and (4) omapatrilat is a relatively mature NEP inhibitor, and the resolution of the crystal is 1.75, which is relatively low.

### 2.7. Statistical Analysis

ANOVA was conducted in comparing different groups containing multiple subgroups. *P* < 0.05 was considered as significant.

## 3. Results

### 3.1. Identification of Key miRNAs

**Figure 1:**
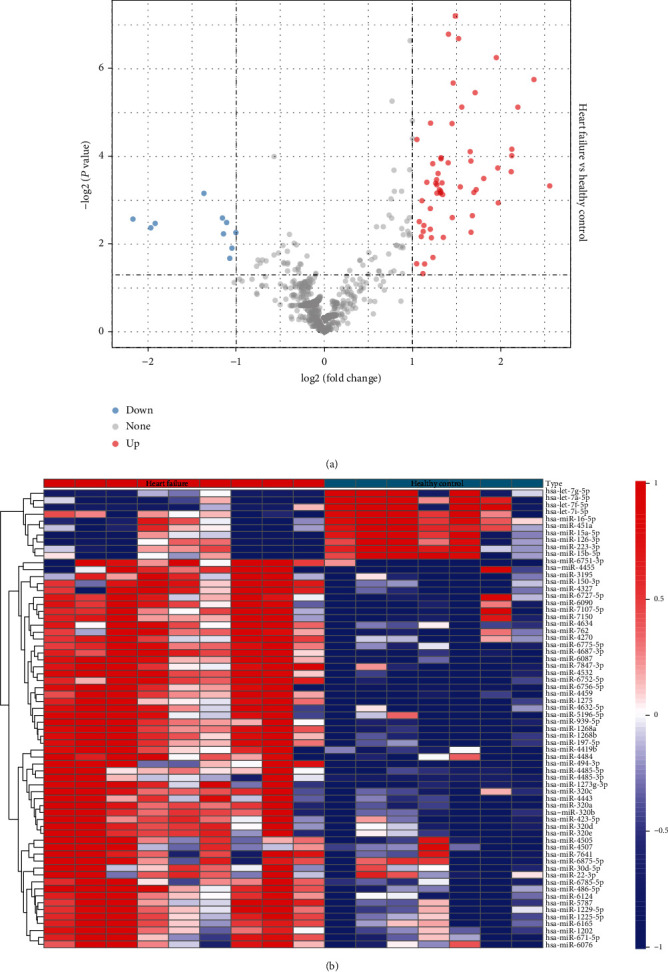
Differentially expressed miRNA analysis on the GSE104150 dataset: (a) volcano plot of miRNA differential analysis; (b) heat map of differential miRNA expression.

First, we identified 66 differentially expressed miRNAs, of which 10 miRNA expressions were downregulated and 56 miRNA expressions were upregulated (Figure 1(a)). Then, we looked at the clustering of the 66 differentially expressed miRNAs and found that the HF group showed significantly different miRNA expression patterns than the control group (Figure 1(b)). To further investigate the functions of the 66 differential miRNAs, we obtained 66 miRNA key target genes, among which hsa-miR-126-3p did not have a corresponding target gene. KEGG enrichment analysis on the target genes showed that the target genes were enriched to the MAPK signaling pathway, TGF-beta signaling pathway, p53 signaling pathway, Prolactin signaling pathway, and JAK-STAT signaling pathway (Supplementary Figure 1).

### 3.2. Identification of Key Target Genes of Key miRNAs

**Figure 2:**
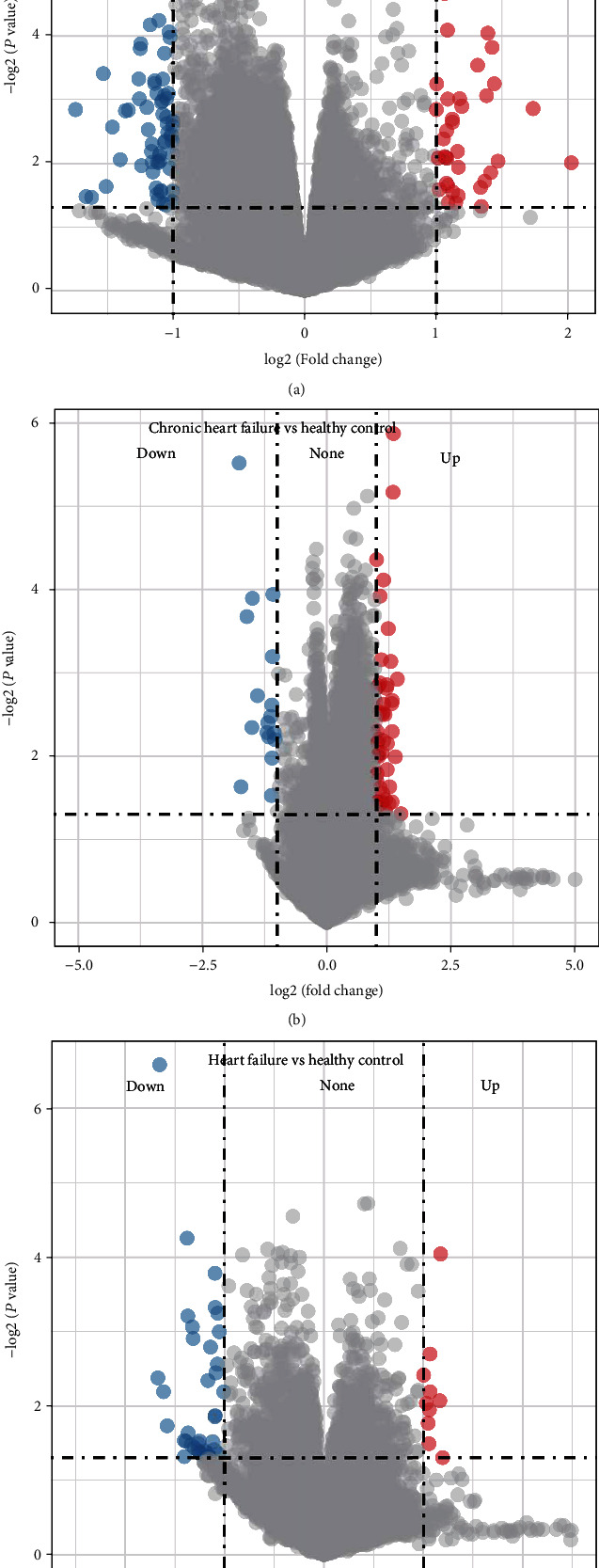
Differentially expressed gene analysis on the GSE21125 dataset: (a) volcano plot of differential analysis of acute heart failure patients vs. healthy individuals; (b) volcano plot of differential analysis of chronic heart failure patients vs. healthy individuals; (c) volcano plot of differential analysis of heart failure patients vs. healthy individuals.

**Figure 3:**
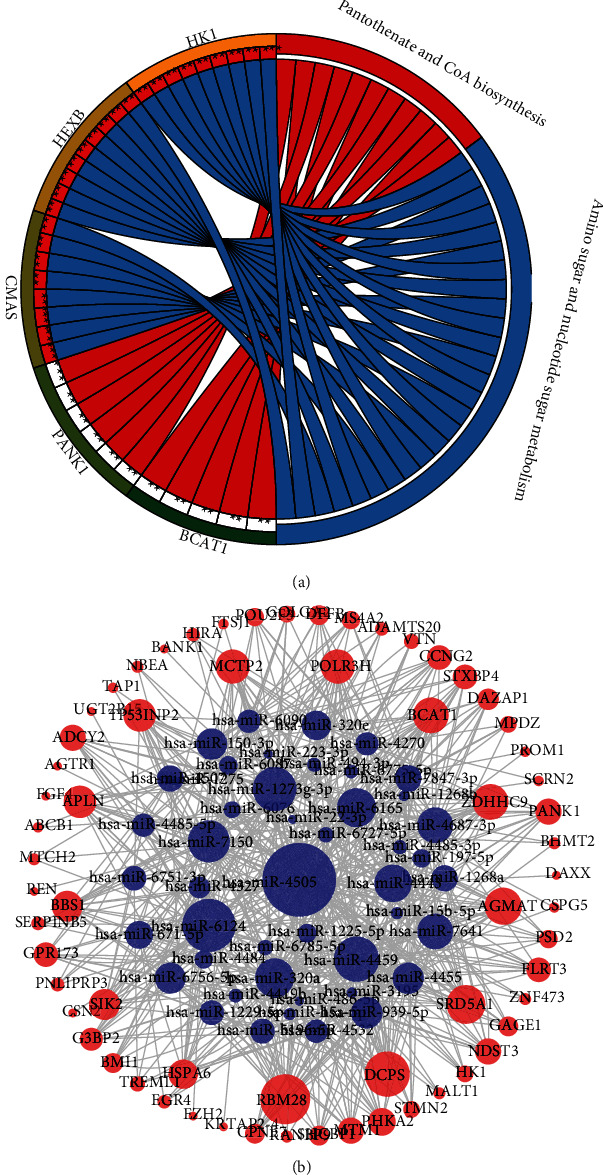
Functional enrichment analysis: (a) KEGG enrichment analysis of differential genes; (b) network plot of miRNA interactions with differential target genes, where blue is miRNA, red is gene, and the size of the dot is degree, where the larger the dot, the closer the connectivity with other nodes at that point, and the more important the dot is.

To identify key genes associated with HF, we performed differential analysis of mRNA expression profile data (HF patients vs. healthy individuals). First, the differential analysis was performed for acute HF patients/chronic HF patients/HF patients vs. healthy individuals, respectively, and we found that 64 genes with downregulated expression and 36 genes with upregulated expression were obtained from the acute HF patients vs. the healthy group (Figure 2(a)); 17 genes with downregulated expression and 48 genes with upregulated expression were obtained from the chronic HF patients vs. the healthy group (Figure 2(b)). After combining chronic HF/acute HF patients with the healthy group for differential analysis, 38 genes with downregulated expression and 10 genes with upregulated expression were finally screened (Figure 2(c)). By combining these differential genes of three groups, we finally found 83 genes upregulated and 92 genes downregulated in HF patients vs. healthy individuals. Next, we performed KEGG enrichment analysis on 175 differential genes (*P* < 0.05). These genes were enriched to a total of two pathways (Figure 3(a)), including amino sugar and nucleotide sugar metabolism, pantothenate, and CoA biosynthesis. Combined with the 66 key differentially expressed miRNAs screened in the above analysis, we found that only 68 of these 175 differential genes were the target genes of these key miRNAs, and subsequently, we constructed a miRNA-mRNA regulatory network, which included 46 miRNAs such as hsa-miR-4505, hsa-miR-6124, and hsa-miR-4459 and 64 miRNAs such as RBM28, DCPS, SRD5A1, and HSPA6 mRNAs (Figure 3(b)).

### 3.3. Pathway Characteristics of Abnormal HF Regulation

**Figure 4:**
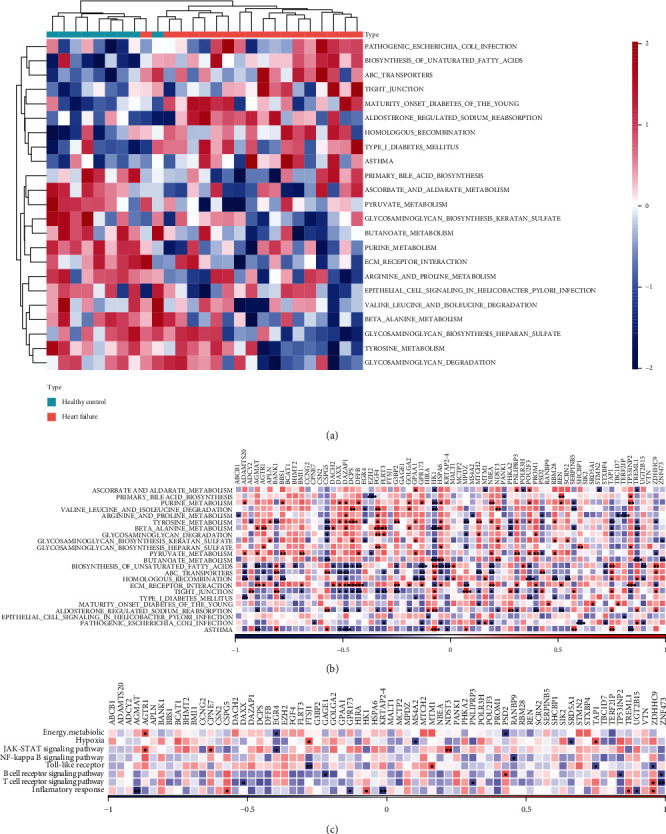
Characterization of pathways abnormally regulated in heart failure: (a) heat map of enrichment scores of pathways significantly different in heart failure patients and healthy group by GSVA (*P* < 0.05); (b) heat map of correlation analysis between related pathways and differentially miRNA-regulated differential target genes; (c) heat map of correlation between energy metabolism, hypoxia score, and inflammation-related pathways and differentially miRNA-regulated; (d) heat map of correlation analysis between energy metabolism, hypoxia score, and inflammation-related pathways and differential miRNA-regulated target genes.

To further understand the abnormal regulatory pathways in the organism triggered by HF, we performed ssGSEA of KEGG-related pathways and found that 23 pathway scores were significantly different between HF patients and healthy groups (Figure 4(a)), including TIGHT_JUNCTION, PATHOGENIC_ESCHERICHIA_COLI_ INFECTION, ABC_TRANSPORTERS, BIOSYNTHESIS_OF_UNSATURATED_FATTY_ACIDS, and MATURITY_ONSET_DIABETES_OF_THE_YOUNG. To further investigate the pathways potentially regulated by the differential target genes of these differential miRNAs, we performed Pearson correlation analysis using the rcorr function of Hmisc package on the expression of these target genes and the 23 pathways (Figure 4(b)). We found that most of the target genes were significantly associated with ECM_RECEPTOR_INTERACTION, ABC_ TRANSPORTERS, HOMOLOGOUS_RECOMBINATION, and BETA_ALANINE_METABOLISM.

HF leads to hypoxia [26, 27]; therefore, we calculated the hypoxic score of each sample in the GSE21125 dataset by the ssGSEA method using genes in the HALLMARK_HYPOXIA pathway as hypoxic key genes, and the correlation between target genes and hypoxic score was determined by Pearson's method. The results revealed that the FTSJ1 gene and TBC1D7 gene were significantly and positively correlated with hypoxia score and that MS4A2 and SRD5A1 genes were significantly and negatively correlated with hypoxia score.

HF not only leads to hypoxia but also may affect the process of energy metabolism of the body. The corresponding scores of genes in these pathways SULFUR_METABOLISM, OXIDATIVE_PHOSPHORYLATION, and NITROGEN_METABOLISM were calculated by ssGSEA, the mean of which was taken as the score to indicate energy metabolism. We found a significant positive correlation between AGTR1 and energy metabolism and a significant negative correlation between EGR4 and PSD2 genes and energy metabolism by the correlation analysis.

Further, we analyzed HF and inflammation [28]. Inflammation-related pathways are Toll-like receptor (TLR) pathway, T cell receptor signaling, NF-*κ*B signaling, Jak/Stat signaling, B cell receptor signaling, and IL-6 receptor family. For the genes in the above related pathways, we calculated their related pathway scores by the ssGSEA method and found that a total of 24 genes were significantly associated with inflammation-related scores, hypoxia-related scores, and energy metabolism-related scores by correlation analysis (Figure 4(c)). These 24 genes were AGMAT, AGTR1, CPNE7, CSPG5, DAXX, EGR4, FTSJ1, GAGE1, GPR173, HK1, KRTAP2-4, MS4A2, MTM1, NDST3, PNLIPRP3, PSD2, RANBP9, SRD5A1, TAP1 TP53INP2, TREML1, UGT2B15, ZDHHC9, and ZNF473.

**Figure 5:**
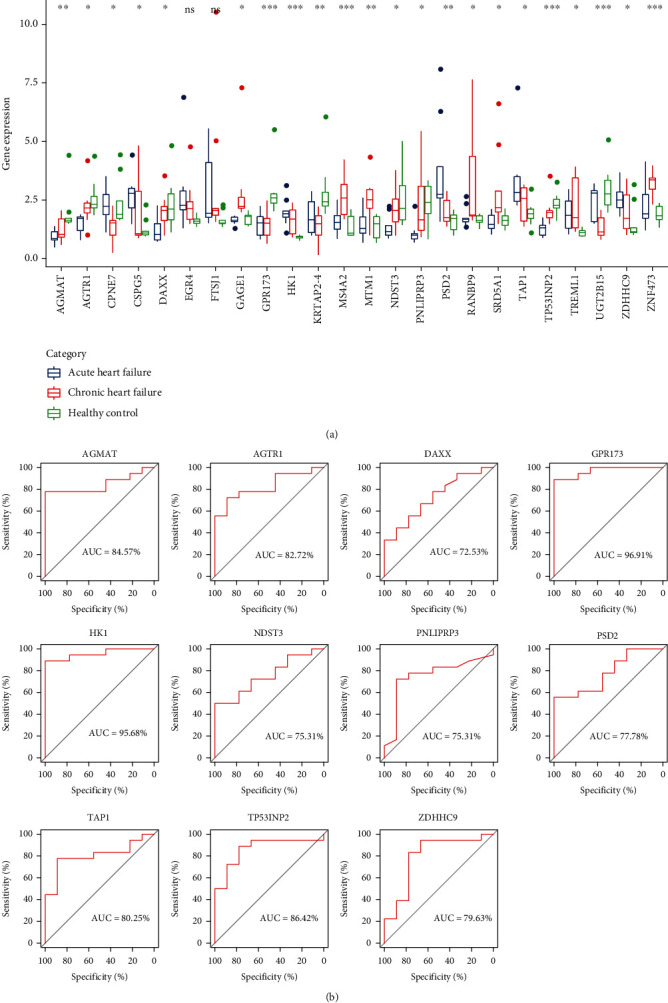
Screening analysis of correlated target genes: (a) box line plot of 24 genes expressed in acute, chronic heart failure and healthy groups (ANOVA, ^∗^*P* < 0.05; ^∗∗^*P* < 0.01; ^∗∗∗^*P* < 0.001; and ^∗∗∗∗^*P* < 0.0001); (b) construction of diagnostic models for 11 key genes.

Through the previous analysis, we found a total of 24 genes significantly associated with inflammation-related scores, hypoxia-related scores, and energy metabolism-related scores. First, we looked at the expression of these 24 genes in HF and healthy groups. The expression of 11 genes, AGMAT, AGTR1, DAXX, GPR173, HK1, NDST3, PNLIPRP3, PSD2, TAP1, TP53INP2, and ZDHHC9, was significantly different between the acute and chronic and healthy groups, while their expression was significantly different in acute-chronic-normal with gradual increase/decrease (Figure 5(a)). We then constructed diagnostic models for these 11 genes and found that the AUC of all the 11 genes reached above 0.7 (Figure 5(b)).

### 3.4. Identification of Key miRNA-mRNA

**Figure 6:**
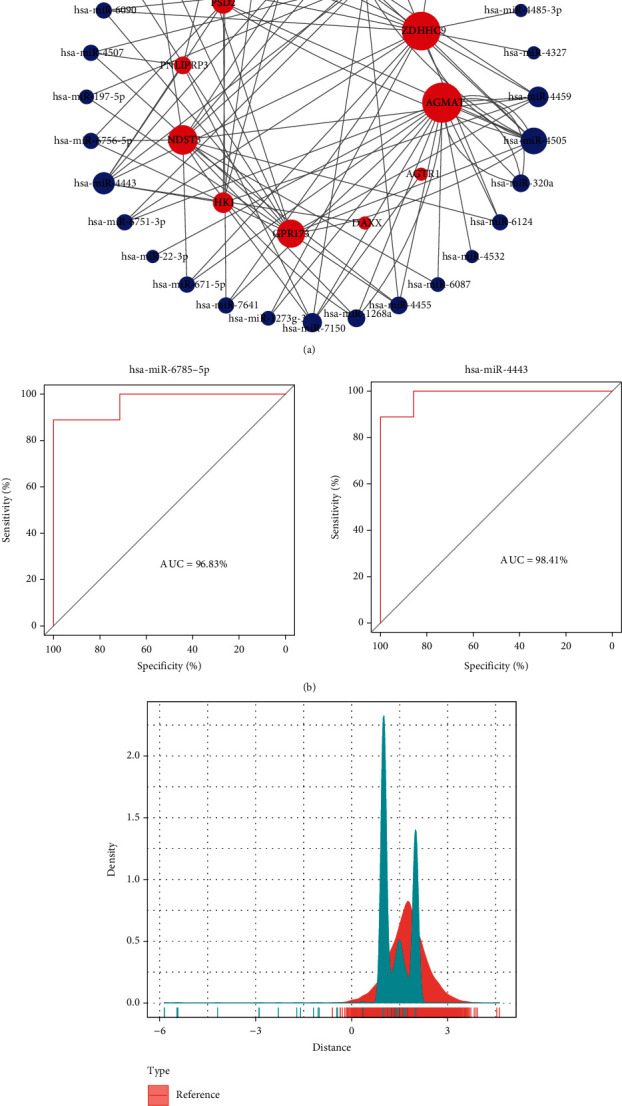
miRNA-mRNA relationship analysis: (a) miRNA-mRNA interaction relationship network; (b) diagnostic model of miRNA; (c) distance density fractionation plot of drug to TAP1-related gene set.

We constructed miRNA-mRNA interaction network based on the 11 key target genes, and through using Cytoscape software [29], it has been found that these 11 target genes had closely related interactions with 32 miRNAs in the miRNA-mRNA network (Figure 6(a)). Among them, four genes were highly expressed in the disease group, namely, ZDHHC9, PSD2, HK1, and TAP1. Next, we explored the crystal structures of the proteins corresponding to these four genes, among which ZDHHC9 and PSD2 showed no crystal structures, HK1 had complete crystals but no one has performed molecular docking on this protein with small molecules so far, and TAP1 had crystals [30, 31], and there are articles on its molecular docking [32]. Therefore, we selected the TAP1 gene as a candidate gene, which had the highest expression in acute HF, followed by chronic HF, and the lowest expression in the healthy group.

Then, based on the miRNA-mRNA interaction network analyzed above, it could be seen that the TAP1 gene was regulated by two miRNAs, hsa-miR-6785-5p and hsa-miR-4443. Next, we constructed diagnostic models for these two miRNAs based on the pROC package and found that the AUC of these two miRNAs reached above 0.9 (Figure 6(b)).

### 3.5. Prediction of TAP1-Related Gene Set and Potential Target Therapeutic Agents

We performed correlation analysis of the genes in the GSE21125 dataset by the rcorr function of the Hmisc package and obtained a total of 119 genes that were highly significantly associated with the TAP1 gene after screening the genes with a correlation greater than 0.4 (*P* < 0.001). We concluded that the above TAP1-related gene sets were important genes for the treatment of HF and that drugs targeting these genes could have a greater impact on HF treatment development. Based on the drug target pairs in DrugBank and the predicted PPI interactions, we calculated the proximity of drugs and the effect on treating HF (Figure 6(c)) and then analyzed the obtained TAP1-related gene set of relevant drug candidates.

**Figure 7:**
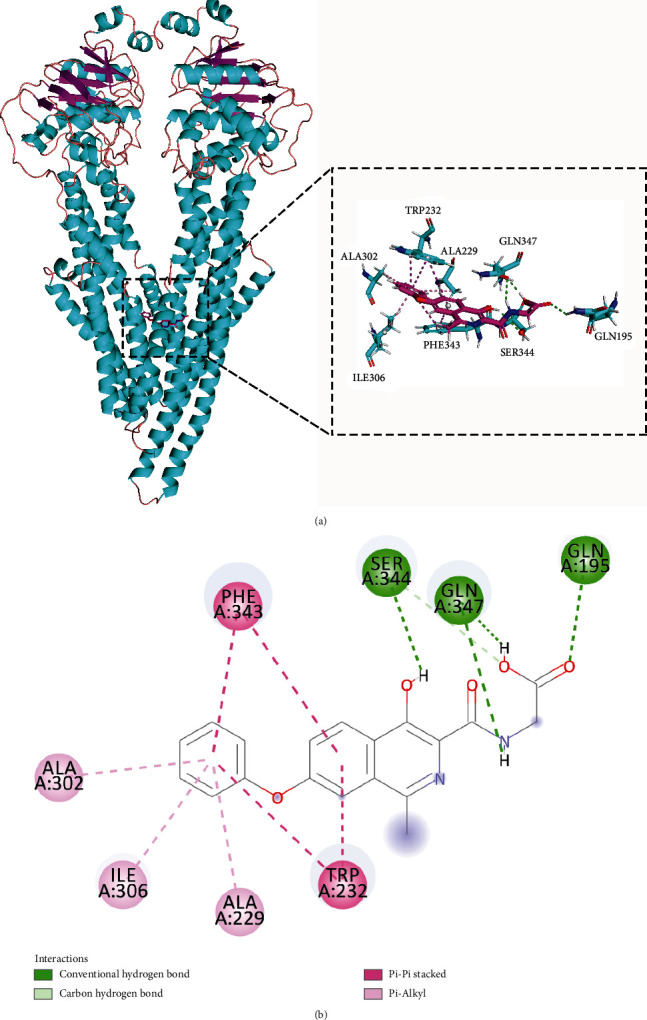
Binding pattern map of TAP1 protein with compound DB04847: (a) 3D binding pattern map of compound DB04847 with TAP1 protein; (b) 2D analysis map of detailed interaction generated by compound DB04847 with TAP1 protein, in which the *α*-helix of the protein backbone is shown as a cyan band and the *β*-fold is shown as a magenta band. Compound DB04847 is shown as a plum-red stick, the amino acid residues that produce the interaction are shown as cyan sticks, and the colors of the heteroatoms in the compound and amino acid residues are shown by element type. Hydrogen bonds are shown as green dashed lines, *π*-*π* stacked interactions are shown as magenta dashed lines, and *π*-Alkyl interactions are shown as pink dashed lines.

We analyzed the potential target compounds by molecular docking and observed that five compounds with TAP1 scored high molecular docking (Table 1) and generated more favorable interactions. Notably, DB04847 had the highest molecular docking score of -9.8 kcal/mol. DB04847 bound in the active site of the TAP1 protein and produced hydrogen bonding interactions with GLN195, SER344, and GLN347 in the binding pocket and generated *π*-Alkyl interactions with ALA229, ALA302, and ILE306 and *π*-*π* stacked interactions with TRP232 and PHE343 (Figures 7(a) and 7(b)). The relatively high molecular docking score and the ability of compound DB04847 to produce so many favorable interactions with TAP1 protein suggested that this compound was highly likely to be a potential inhibitor of TAP1.

### 3.6. Pathways Abnormally Regulated by the TAP1 Gene

**Figure 8:**
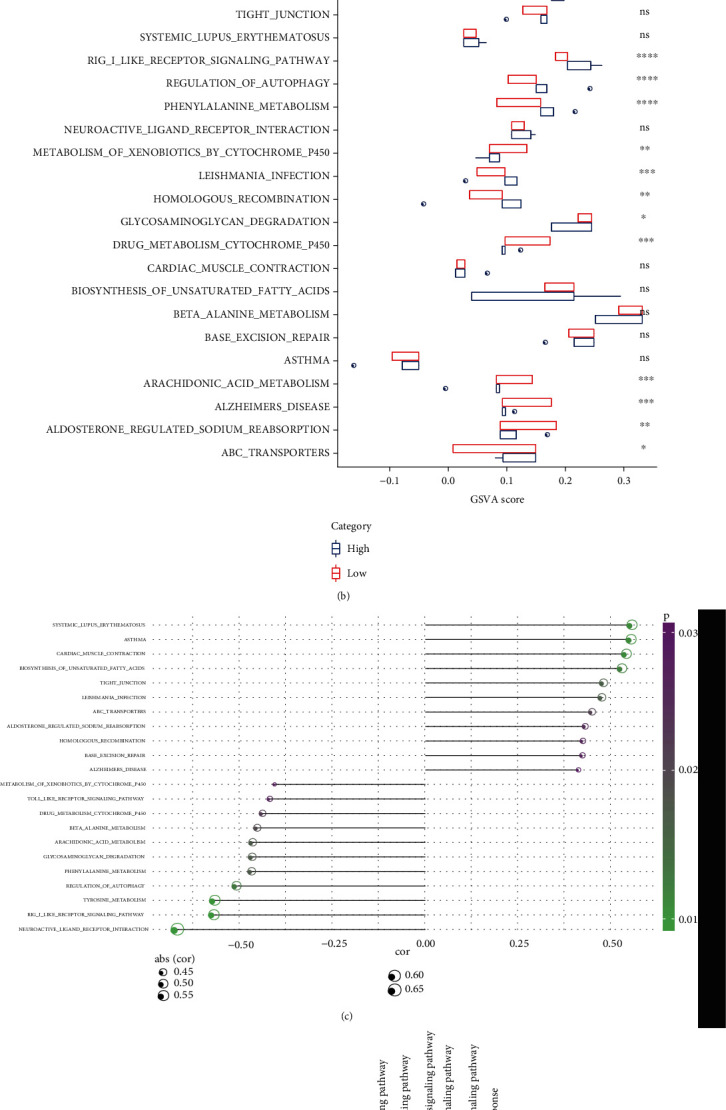
Pathway analysis of aberrant regulation of TAP1 gene: (a) heat map of aberrantly regulated pathway enrichment scores; (b) difference in aberrantly regulated pathway enrichment scores between the high- and low-expression groups of the TAP1 gene; (c) point bar graph of correlation between aberrantly regulated pathway enrichment scores and TAP1 gene expression, where color is significance and size of point is strength of correlation; (d) correlation between TAP1 expression and hypoxia, energy metabolism, and inflammation-related pathways in a correlation heat map.

To better investigate the pathways potentially regulated by the TAP1 gene, we screened a total of 22 TAP1 aberrantly regulated pathways by enrichment score and TAP1-pathway correlation (Figure 8(a)), and we also divided the TAP1 gene into high- and low-expression groups by median value. It has been found that 14 of these 22 aberrantly regulated pathways (64%) were significantly different in the high- and low-expression groups (*t*-test.  Figure 8(b)). We further looked at the correlation of these 22 aberrantly regulated enrichment scores with TAP1 expression (Figure 8(c)). SYSTEMIC_LUPUS_ERYTHEMATOSUS, ASTHMA, CARDIAC_MUSCLE_CONTRACTION and BIOSYNTHESIS_OF_UNSATURATED_ FATTY_ACIDS, LEISHMANIA_INFECTION, and ABC_TRANSPORTERS pathways had enrichment scores positively correlated with TAP1 expression, and NEUROACTIVE_LIGAND_RECEPTOR_INTERACTION, RIG_LIKE_RECEPTOR_SIGNALING, and TYROSINE_METABOLISH had enrichment scores negatively correlated with TAP1 expression. In addition, correlation analysis of energy metabolism, hypoxia, and inflammation-related pathways with TAP1 expression showed a significant positive correlation between TAP1 and hypoxia pathway and a significant negative correlation with Toll-like receptor signaling pathway (Figure 8(d)). The above data indicated that TAP1 was closely correlated with classic cell growth pathways.

## 4. Discussion

HF is a globally common clinical syndrome characterized by structural damage to the heart and/or cardiac dysfunction leading to fatigue at rest and dyspnea [33]. HF is a multifactorial disease, the development of which is associated with complex regulation. Despite numerous studies [34, 35], the exact mechanisms of HF remain to be elucidated in order to facilitate the discovery of key drug targets in HF [36]. In this study, we screened miRNAs associated with HF by differential analysis and obtained miRNA-regulated mRNAs (genes) and explored the pathway characteristics of abnormal regulation of HF by enrichment analysis of miRNA target genes.

Our analysis revealed a significant positive correlation between TAP1 and hypoxia score, which is closely associated with HF [26, 27]. TAP1 belongs to the ATP binding cassette (ABC) transporter protein superfamily [37]. The existence of ABC_TRANSPORTERS in the regulatory pathways involved in the differential target genes of HF-related miRNAs is consistent with the above results. Moreover, TAP1 is mainly involved in transporting antigen from the cytoplasm to the endoplasmic reticulum, binding to major histocompatibility complex (MHC) class I molecules, and acting as a molecular scaffold for the final stage of MHC class I folding, that is, peptide binding [38]. Thus, TAP1 can perform antigen-presenting functions and regulate adaptive immunity [39]. TAP1 has been reported to be associated with tumor immune escape, and high-expressed TAP1 has been seen as a poor prognostic factor in stage I/II colorectal cancer patients [40]. In the present study, TAP1 was highly expressed in HF patients, which also suggests that high TAP1 expression is an unfavorable factor in the disease of HF. There is no reported association of TAP1 with the mechanism or prognosis of HF other than the present study, much less a study reporting TAP1 as an important gene for HF treatment. Therefore, for the first time, this study revealed the important function of TAP1 in HF management, and molecular docking verified that DB04847 was a potential inhibitor of TAP1.

Two key miRNAs, hsa-miR-4443 and hsa-miR-6785-5p, were recruited into the diagnostic model of miRNAs regulating TAP1 gene constructed in this study. hsa-miR-6785-5p was reported to be a novel target for diagnosis of advanced bladder cancer and its prognosis. It has been reported that LINC01929, which is highly expressed in advanced bladder cancer, upregulates the expression level of ADAMTS12 through competitive adsorption of miR-6875-5p, and based on this molecular mechanism, overexpressed miR-6875-5p inhibits the progression of bladder cancer [41]. However, there are no reports on hsa-miR-6785-5p in HF. Some other studies showed that miR-4443 can inhibit metastasis and energy metabolism of papillary thyroid cancer through targeting TRIM14 [42]. Most importantly, it has been reported that hsa-miR-4443 is implicated in atrial fibrillation regulation; that is, in atrial fibrillation, hsa-miR-4443 regulates TGF-*β*1/*α*-SMA/collagen signaling via targeting THBS1, thereby inhibiting cardiac fibroblast proliferation [43]. This study supported the involvement of hsa-miR-4443 as a potentially important miRNA in HF and as a potential target for HF therapy.

Although many adequate analyses have been conducted earlier in this paper, our study still has several limitations. First, the sample size of the current work was small; therefore, a larger cohort to further validate these results is required. Secondly, the specific biological functions of miRNAs in diagnostic models are still unclear, and whether these miRNAs could exert regulatory effects on pathways implicated in HF requires future exploration. Because HF is a heterogeneous syndrome that mainly affects patients suffering from multiple comorbidities, it is not uncommon that there are some overlaps in the mechanisms of other diseases; thus, the pathways identified in this study could be as well important in associated comorbidities. For further overcoming the limitations of this study, we are planning to re-collect and expand the clinical sample in subsequent work and will validate the accuracy of this drug target and model through additional external experiments.

Our analysis of the GEO dataset provided drug targets and diagnostic models for HF management. The drug target and model provide a comprehensive perspective to study the prognostic features and treatment of HF, and the newly discovered TAP1-mediated miRNA-regulated diagnostic model may provide new insights into the current knowledge of the mechanisms of HF initiation and progression as well as a new idea and basis for further study of HF treatment options.

## 5. Conclusion

In the treatment of HF, we identified TAP1 as a potential target and predicted that DB04847 drug is highly likely to be a potential inhibitor of TAP1. In addition, two miRNAs (hsa-miR-6785-5p and hsa-miR-4443) that regulate TAP1 targets have a potential diagnostic value.

## Figures and Tables

**Table 1:** Molecular docking scores of compounds with TAP1 proteins and generated important interactions.

Compound	AutoDock Vina score	H-bond interactions	Hydrophobic interactions
DB04847	-9.8	GLN195, SER344, GLN347	ALA229, TRP232, ALA302, ILE306, PHE343
DB01116	-8.7	GLN990	ALA229, TRP232, ILE306, LEU339, ILE340, PHE343
DB06412	-8.1	ASN721, GLN838	PHE303, TYR307, ALA987, VAL991
DB00480	-7.9	GLN990	TRP232, PHE343
DB08378	-7.6	TRP232, ASN721, GLY722, SER766, ASN842	PHE303, VAL991

## Data Availability

The data used to support the findings of this study are included within the article.

## Supplementary Materials

Supplementary Materials Supplementary Figure 1: functional enrichment of differential miRNAs.
