# Supplementary figures and images for: Mining Potential Drug Targets and Constructing Diagnostic Models for Heart Failure Based on miRNA-mRNA Networks
Source: Mediators Inflamm. 2022 Sep 27;2022:9652169. doi: 10.1155/2022/9652169 (PMC9532133; doi:10.1155/2022/9652169)

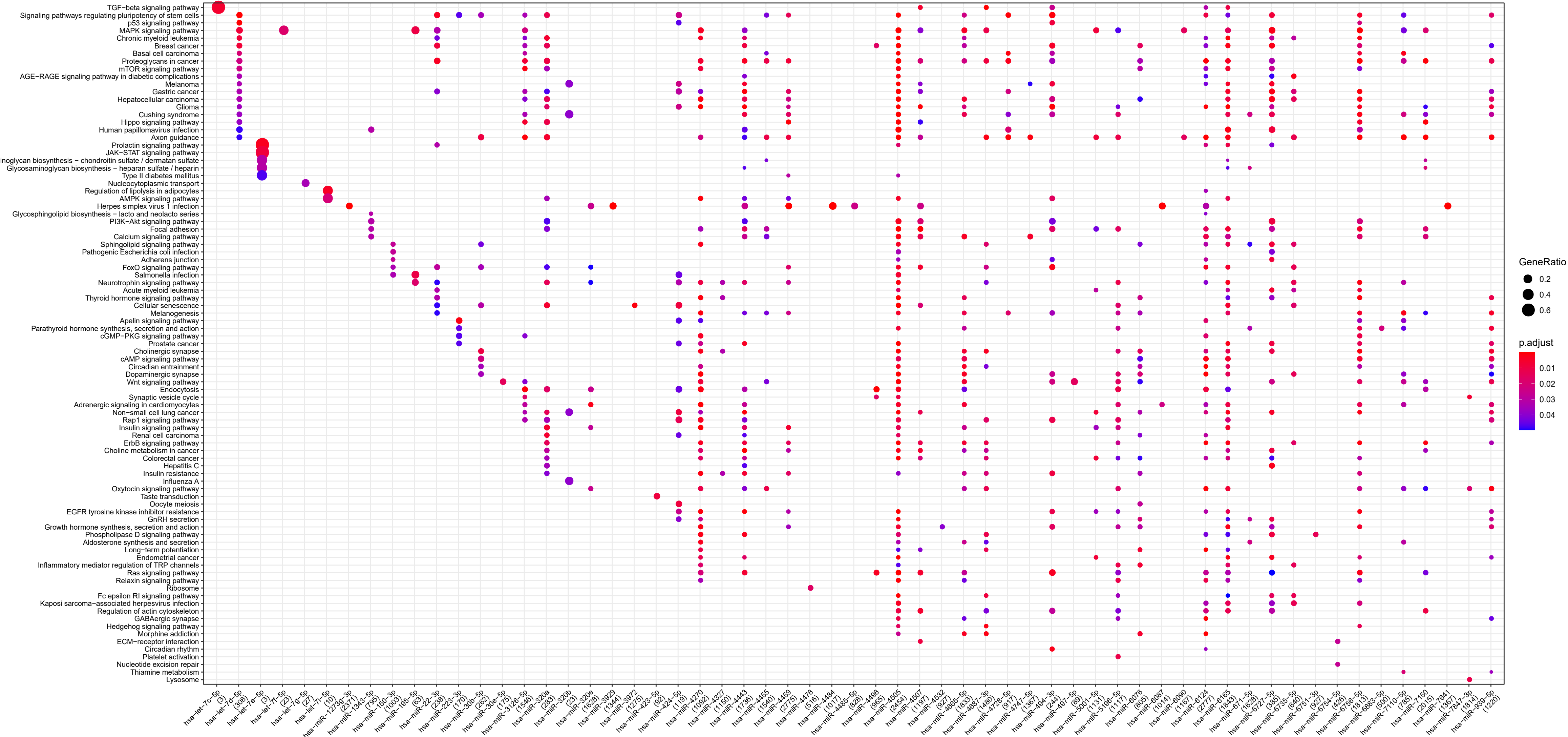

Supplement: Supplementary Materials — Supplementary Figure 1: functional enrichment of differential miRNAs. [file 9652169.f1.pdf]
